# Oligotrophy vs. copiotrophy in an alkaline and saline habitat of Lonar Lake

**DOI:** 10.3389/fmicb.2022.939984

**Published:** 2022-08-04

**Authors:** Yogesh S. Nimonkar, Tejashree Godambe, Apurva Kulkarni, Tarachand Patel, Dhreej Paul, Debarati Paul, Vinay Rale, Om Prakash

**Affiliations:** ^1^National Centre for Microbial Resource, National Centre for Cell Science, Pune, India; ^2^Amity Institute of Biotechnology, Amity University Uttar Pradesh (AUUP), Noida, India; ^3^Symbiosis School of Biological Sciences (SSBS) Symbiosis International (Deemed University) & Symbiosis Centre for Research & Innovation (SCRI), Symbiosis International (Deemed University), Pune, India

**Keywords:** nutrition, growth-rate, microbial-ecology, industrial enzymes, nutrient cycling, lifestyle switch

## Abstract

We reported our comparative observations on oligotrophs vs. copiotrophs from a hyper-alkaline and hypersaline habitat, Lonar Lake, situated in the Buldhana district of Maharashtra, India. Cell numbers of oligotrophic and copiotrophic microbes from the sediment were enumerated by the three-tube most probable number (MPN) method using an array of nutrient-rich and oligotrophic (≈10–20 mg carbon L^−1^) media offering simulated natural conditions of pH and salinity. A total of 50 strains from 15 different genera and 30 different species were isolated from the highest positive dilutions of MPN to identify the taxa of oligotrophs and copiotrophic microorganisms dominating in Lonar Lake. We did not get any true oligotrophs due to their adaptation to higher carbon levels during the isolation procedure. On the contrary, several true copiotrophs, which could not adapt and survive on a low-carbon medium, were isolated. It is also observed that changes in medium composition and nutrient level altered the selection of organisms from the same sample. Our data indicate that copiotrophic microorganisms dominate the eutrophic Lonar Lake, which is also supported by the past metagenomics studies from the same site. We also reported that quick depletion of carbon from oligotrophic medium worked as a limiting factor, inducing cell death after 2–3 generations and preventing the development of visible colonies on plates and sufficient optical density in liquid medium. Therefore, a long-term supply of low levels of carbon, followed by isolation on enriched media, can serve as a good strategy in isolation of novel taxa of microorganism, with industrial or environmental importance.

## Highlights

- The composition and concentration of culture media play an essential role in the selection of microorganisms.
- A continuous supply of low nutrient levels for the long term is imperative to enrich the oligotrophic microbes.
- Isolated oligotrophs with valuable enzyme production potential can be a valuable resource for cost-effective industrial enzyme production.
- Oligotrophs from this study can serve as a tool for OMICS studies to understand microbes' lifestyle and survival tactics under ultra-low nutritional support and can extend this experience to other comparable situations.

## Introduction

Microbes have adapted to various growth and survival strategies to survive and reproduce. Ecologically challenging habitats with varying nutrient availability impose intense selective pressure on microbes that determine their lifestyle tactic (Lauro et al., 2009). The response of bacteria to carbon input in a particular ecological niche helps to understand the population dynamics of microbes and their interactions with other biotic and abiotic components (Watve et al., 2000; Shahbaz et al., 2020).

Depending on their ability to adapt to various substrate concentrations, especially carbon, microbes are classified as oligotrophs and copiotrophs (Koch, 1998; Saha and Chakrabarti, 2006; Ohhata et al., 2007). The bacteria able to grow on low nutrient concentrations but unable to survive in nutrient-rich conditions, especially high load of carbon, are known as “oligotrophic bacteria” (Hu et al., 1999; Koch, 2001; Ohhata et al., 2007). Oligotrophs can be designated as “obligates” when they strictly require low concentrations of carbon for growth and “facultative” when they grow in low as well as high levels of carbon (Ishida and Kadota, 1981). Slow growth and metabolic rates, higher substrate affinity, and generally low population density are the characteristics of oligotrophic organisms (Ho et al., 2017). These properties allow them to preponderate in natural habits with limited available/utilizable carbon (Amy and Morita, 1983; Morita, 1988; Fegatella and Cavicchioli, 2000; Ferrari et al., 2005; Temperton et al., 2011).

In contrast, copiotrophs predominate nutrient-rich environments such as sewage lagoons, carbon-rich soils, and decomposing wood. They are adapted to use carbon sources rapidly and are especially suited to habitats with high nutrient flux (Fry, 1990). Thus, the oligotrophs have a competitive advantage over copiotrophs and can flourish in nutrient-limited systems (Button, 1991). Most aquatic and terrestrial habitats are nutrient-depleted and oligotrophic.

Habitat condition, nutrient availability, biodiversity, and unique adaptability (Paul and Clark, 1996) are the key factors influencing the plethora of microbes having the necessary and varied ability for carbon utilization. Oligotrophic and copiotrophic strategies give an insight into the adaptations and strategies employed by microorganisms in response to different nutrient requirements. Thus, studying contrasting survival strategies of both oligotrophs and copiotrophs is essential to track the microbial evolution and for an in-depth understanding of the two modes of livelihood or survival (Koch, 2001). An improved understanding of trophic strategies is also essential to understand the biogeochemical cycles and microbial responses to changes in the bioavailability of resources (Antony et al., 2013). From a historical perspective, it is interesting to note that the development of nutrition strategies for isolation has undergone sea change from the methodologies of the “Delft School” (liberal nutrition) to the demise of the concept of autotrophy *sensu stricto* (autotrophs shown to use organic carbon) (Kelly, 1971).

Lonar Lake, a hypersaline and hyper-alkaline soda lake located in Buldhana district, Maharashtra, India, harbors a unique ecological niche. It is the only crater formed due to the high-velocity meteoritic impact on basaltic rock. Many studies have reported the biodiversity associated with Lonar Soda Lake (Joshi et al., 2007; Pathak and Deshmukh, 2012; Tambekar and Dhundale, 2012; Sharma et al., 2016). Microbes isolated from the soda lake region manifest complex microbial food webs interconnecting various biological cycles *via* redox coupling. The inhabitants have been reported to possess biotechnologically important enzymes and biomolecules (Hinder et al., 1999; Antony et al., 2010; Grant and Sorokin, 2011). However, studies on the trophic interactions between these microbes and their role in the ecosystem are not adequately documented. The microbial diversity of such ecosystems has the potential to throw light on adaptability to extreme conditions and possibly pave the way to hunt for novel and industrially valuable biomolecules. In this study, we have cultured, quantified, identified, and metabolically characterized the oligotrophs and copiotrophs isolated from the hypersaline Lonar Lake sediment samples. In addition, we also tried to investigate the strategy that is prevalently used by indigenous organisms to survive in a unique extreme habitat such as Lonar Lake.

## Materials and methods

### Site description and sampling

Sediment samples used for cultivation and enumeration studies were collected from the hyper-alkaline and hypersaline Lonar Lake located in Buldhana district, Maharashtra, India (19°59′N, 76°31′E), using the grab sampling method. The sampling site's geographic location and physiochemical features are described anywhere else (Joshi et al., 2008; Antony et al., 2010, 2013, 2014; Surakasi et al., 2010; Borul, 2012; Paul et al., 2016; Bagade et al., 2020). For microbiological investigation, sediment samples from different locations of Lonar Lake were aseptically collected in pre-sterilized Falcon tubes, stored immediately on ice, and subsequently transported to the laboratory within 24 h. For cultivation and quantification purposes, samples were stored at 4°C and processed within 4 days.

### Cultivation and enumeration of bacteria

Oligotrophic and copiotrophic bacterial populations from the sample were enumerated by using a three-tube most probable number (MPN) method (Böllmann and Martienssen, 2020). For this, samples were serially diluted using oligotrophic and nutrient-rich growth media. Media such as nutrient broth (NB), Luria broth (LB), tryticase soy broth (TSB), and Ravan and Reasoner's 2A agar (R2A agar) were used for copiotrophs. For oligotrophs NB, LB, and TSB (1,000-fold diluted); (R2A) (100-fold diluted); Ravan (10-fold diluted); and a synthetic medium (fortified with 20 mg carbon L^−1^) were deployed for the cultivation and enumeration of oligotrophic organisms (Tada et al., 1995; Saha and Chakrabarti, 2006; HuiXia et al., 2007; Matsuoka and Yoshida, 2018). Compositions of Ravan and the synthetic groundwater media have been reported previously by Nagarkar et al. (2001) and Green et al. (2010), respectively. Diluted media were supplemented with filter-sterilized vitamins and trace elements (Widdel and Bak, 1992). To mimic the *in situ* pH and salinity conditions of Lonar Lake, medium pH and salinity were adjusted to 9 and 1% (w/v), respectively. Carbon concentration of diluted oligotrophic medium was tentatively estimated by dividing the actual nutrient load of rich medium with dilution factors.

MPN series were prepared in loosely capped tubes and incubated aerobically at 30°C for 72 h in case of copiotrophs and up to 1 month for oligotrophs to grow. Growth of the isolates was monitored visually by observing the change in turbidity compared to uninoculated autoclaved controls. MPN was determined from statistical tables published by the American Public Health Association (https://microbeonline.com/probable-number-mpn-test-principle-procedure-results). For isolation of dominant bacterial groups cultivated in different media, 100 μl aliquots from the highest positive dilutions of MPN series were spread plated on respective media to be incubated at 30°C till the emergence of colonies. Culture purity was obtained by repeated subculturing on respective solid media. Isolated pure cultures were preserved at −80°C with 20% glycerol (Prakash et al., 2013).

### DNA extraction, 16S rRNA gene sequencing, and sequence analysis

Total genomic DNA of all the purified strains was extracted using Purelink-Pro 96-well genomic DNA isolation kit (Invitrogen, USA), following the instructions of the manufacturer. PCR amplification of 16S rRNA gene of the isolated genomic DNA was done using bacteria-specific universal primers 27F (5'-AGAGTTTGATCMTGGCTCAG-3') and 1492R (5'-TACGGYTACCTTGTTACGACTT-3') (Marathe et al., 2013). For 100 μl PCR reaction, 100 ng genomic DNA, 10 μl 10 × buffer, 5 μl MgCl_2_ (25 mM), 2 μl dNTP mix (10 mM), 10 moles of each primer, and 2U of Taq polymerase (Promega, USA) were used. The PCR reaction was done with an initial denaturation condition at 94°C for 5 min; followed by 30 cycles of denaturation at 94°C for 60 s, annealing at 58°C for 45 s, elongation at 72°C for 90 s, and final elongation at 72°C for 7 min. Amplified products were purified by polyethylene glycol method. Thereafter, sequencing was performed using Sanger's method (Big-dye terminator chemistry) as discussed in Prakash and Lal (2013) and Prakash et al. (2014).

The obtained sequences were manually checked for authenticity, and contigs were generated with the SeqMan program of DNASTAR. Identity of isolated pure cultures was determined by similarity search of the obtained sequences using the EzTaxon database (http://www.ezbiocloud.net/eztaxon) (Kim et al., 2013) and BLAST (NCBI) (http://blast.ncbi.nlm.nih.gov/Blast.cgi) program.

### Determination of physiological features of the strains

Physiological parameters, such as range and optima of temperature, salinity, and pH, were determined according to Prakash et al. (2015). Oligotrophic, facultative oligotrophic, and copiotrophic nature of the strains was determined by supplying different concentrations (20, 200, 2,000, 20,000 mg L^−1^) of carbon source to the growth medium with optimum nutrient, pH, and salinity. Plates were incubated at 30°C for 8 days, and growth response of the strains was recorded. It is speculated that during dilution of the complex medium the concentration of key nutrient elements such as carbon, nitrogen, and phosphorus is depleted and may adversely affect growth. To test the effect of nitrogen and phosphorus on bacterial growth, 100 mg L^−1^ NH_4_Cl and K_2_HPO_4_ were added to the diluted medium. Facultative oligotrophs cultivated in a diluted medium were streaked on the plate, and their growth response was evaluated compared to unsupplemented control (Smith and Prairie, 2004).

### Screening for enzyme production

An array of enzymatic activities such as DNase, amylase, protease, urease, cellulase, phosphatase, and gelatinase of the isolated strains was tested using standard protocols described in Smibert and Krieg (1994) unless mentioned otherwise. For DNase activity, bacterial strains were incubated in a medium containing tryptose 20 g L^−1^, toluidine blue 0.1 g L^−1^, and DNA 2 g L^−1^. After 48 h incubation, the plates were flooded with 1 N HCl solution. DNase-positive strains showed a clear zone around the colonies on plates. Amylolytic activity of the strains was tested using a starch agar medium. After 48 h incubation at 37°C, the plates were flooded with 0.6% KI solution, and a clear zone was observed around a positive strain due to the hydrolysis of starch. Proteolytic activities of the cultures were screened using 1% skim milk containing nutrient agar medium. A zone of clearance around the colonies appearing over the next 48 h was considered protease positive. Urease production was screened on *a urea* agar base (Oxoid) supplemented with 40% urea stock. The appearance of a zone of clearance encircling colonies after incubating for 48 h was considered positive. For cellulase production, carboxymethylcellulose agar was used. After 48 h of incubation, we flooded the plates with 0.03% Congo Red to look for a clear zone around the colonies. Screening of phosphatase activity was carried out on Pikovskaya agar medium, where positive strains were determined by visualization of a clear zone after 48 h incubation. Similarly, isolated strains were inoculated in NB with 12% gelatin for the gelatin assay and the organism's ability to liquefy gelatin after 48 h incubation was determined.

### Carbon source optimization

**Figure 1:**
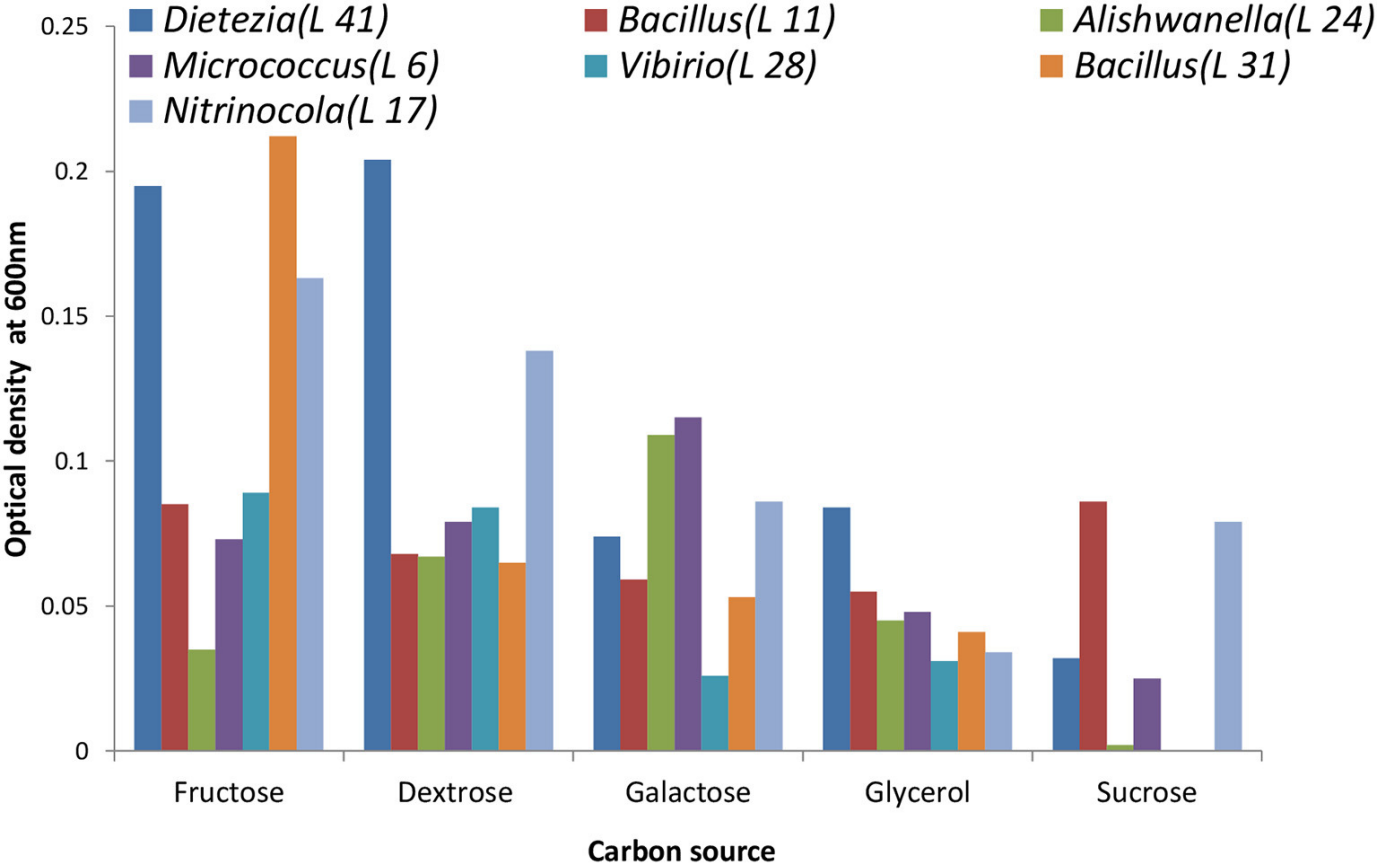
Utilization of different carbon sources by the isolates. Dextrose proved to be the most suitable one for stable growth.

It is recommended that for a comparative study of growth rate and generation time all strains should be cultivated on a common carbon source using the same type of synthetic medium and incubation condition. For that purpose, growth response of selected strains on different sugars (carbon source) was evaluated before the actual experiment. For this purpose, the chosen synthetic groundwater medium (Green et al., 2010) was supplemented with 50 mM of different sugars (dextrose, fructose, galactose, glycerol, and sucrose) as a carbon source, and an increase in optical density at 600 nm was detected using 96-well plate reader Bioscreen-C (Oy Growth Curves Ab Ltd., Finland). In brief, we prepared a synthetic groundwater medium, adjusted the pH to 8 using 1 N NaOH, and sterilized it by autoclaving. The medium was supplemented separately with 50 mM of filter-sterilized different sugars as a carbon source and inoculated with an equal volume of (50 μl) of culture inoculum (OD 0.5 at 600 nm) from all oligotrophic isolates in replicates of three. Plates were incubated in the incubator shaker at 30°C for 48 h at 150 rpm. Growth was monitored at different time points using a spectrophotometer at 600 nm. We then plotted clustered column chart using time and optical density data to determine the growth response of selected oligotrophs on different carbon sources (Figure 1).

### Growth kinetics at different carbon levels

**Figure 2:**
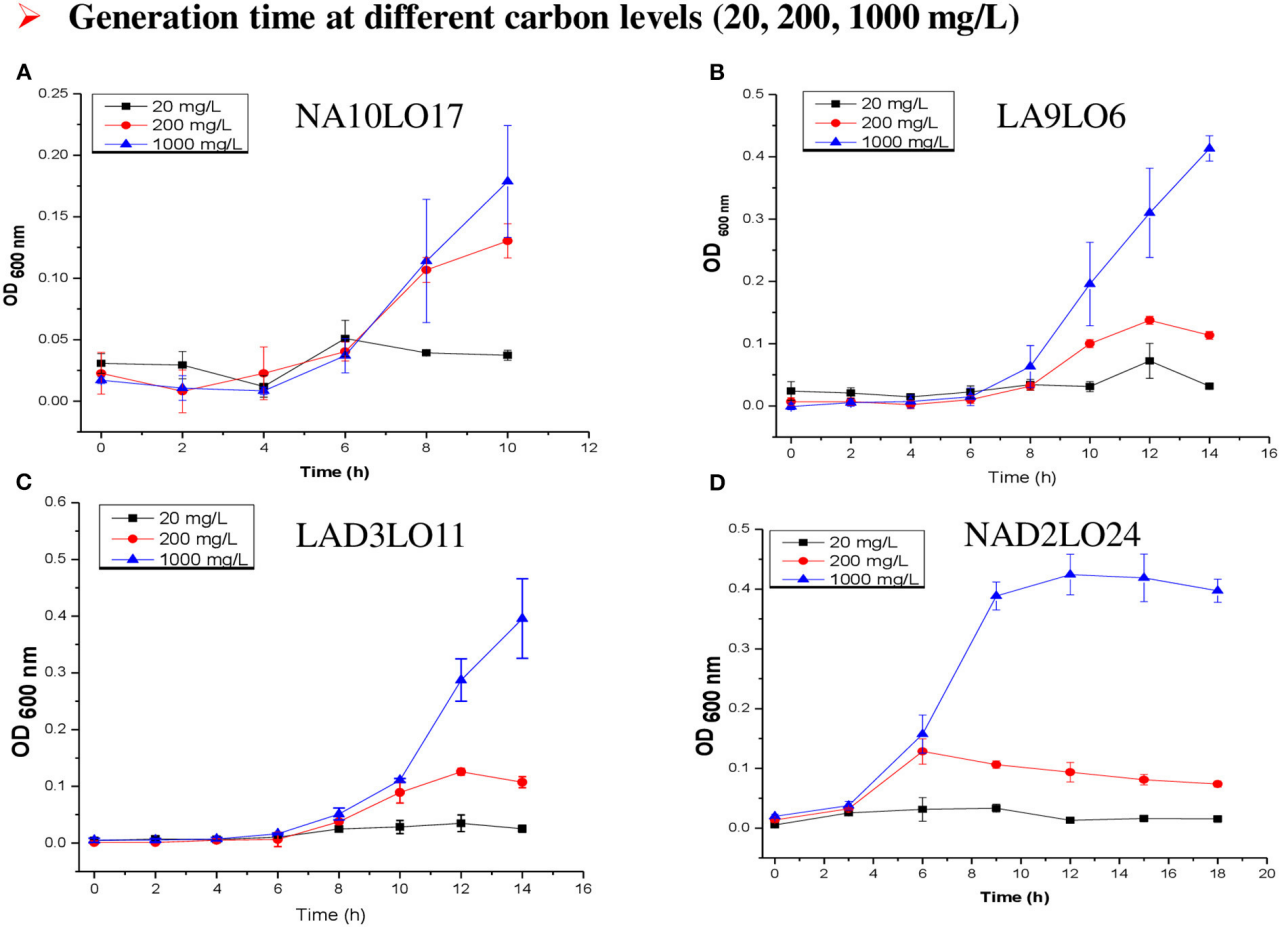
**(A–D)** Growth of **(A)** NA10LO17, **(B)** LA9LO6, **(C)** LAD3LO11, and **(D)** NAD2LO24 in different carbon concentrations (20, 200, and 1,000 mg L^−1^).

Among the tested carbohydrates, dextrose gives a satisfactory growth response to all the selected oligotrophs and was chosen for the growth kinetics experiment. The experiment was conducted in synthetic groundwater medium using the different concentrations of dextrose as sources of carbon and energy and calculated the generation times from obtained optical density or CFU data (Figures 2A–D). In brief, synthetic groundwater medium was prepared and supplemented with different concentrations (20, 200, and 1,000 mg L^−1^) of filter-sterilized dextrose. A volume of 20 ml culture medium was taken in a 100-ml capacity flask, inoculated with 1% inoculum (OD at 600 nm = 0.5), and incubated at 30°C at 150 rpm. Growth at different time intervals (0, 2, 4, 6, 8, …, 24 h) was monitored using a 96-well plate spectrophotometer (at 600 nm). Optical density was plotted against time to calculate the generation time and growth rate. The number of generations (*n*) was calculated using the formula *n* = 3.3 log b/B (“b” represents the optical density at the start of the exponential phase and “B” represents the optical density at the end of the exponential phase). Generation time (G) was calculated using G = t/n, where “t” represents a time period of exponential phase, and “n” represents the number of generations. The growth rate constant was calculated by K = 1/G, where “G” represents generation time. This relationship is valid only during the period when the population is in the exponential phase. Therefore, for different isolates, the “t” varied and the onset of the exponential phase also varied.

### Statistics and sequence accession numbers

All the physiological data related to salinity, pH, and temperature were generated using a minimum of three biological replicates using a similar set of conditions. Experiments related to the screening of extracellular enzyme production were also conducted using multiple replicates, and the reported observations are based on the reproducibility of data. The experiments related to the screening of suitable carbohydrates for growth and growth kinetics were conducted in triplicates. The average and standard deviation of the data were calculated using Excel, the graph was generated in Excel, and the standard deviation in data was presented at each point of the graphs. The sequences retrieved from this study have been deposited in the GenBank database under accession no. ON562524 - ON562538, ON905511, ON905512, ON905513, ON905514, ON905515, ON905516, ON905517, ON905518, ON905519, ON905520, ON905521, ON905522, ON905523, ON905524, ON905525, ON905526, ON905527, ON905528, and ON905529.

## Results

### Isolation, identification, and characterization

As expected, MPN counts of organisms cultivated in nutrient-rich media sets of media doubled are 2-fold higher than those grown in nutrient-deficient media. A total of 49 bacterial strains from the highest positive dilutions of MPN series made in different culture media were isolated in this study. Isolates obtained on different media used are shown in Table 1. Genera such as *Pantoea, Micrococcus, Alishewanella, Nitrincola*, and *Bacillus* were dominant from the highest dilutions (Table 1). 16S rRNA gene sequencing approaches showed that isolated strains belong to 30 different species from 15 genera, including *Bacillus, Exiguobacterium, Dietzia, Staphylococcus, Enterobacter, Vibrio, Penibacillus, Halomonas, Lysinibacillus, Pseudomonas, Pantoea, Alishewanella, Rheinheimera, Micrococcus*, and *Nitrincola*. Eight species of the genus *Bacillus* were detected among the isolated strains, while only 1–3 species of other genera were present. Tables 2, 3 show the strains' taxonomical affiliations and basic physiochemical traits. It was observed that all the isolated species of *Bacillus, Penibacillus*, and *Lysinibacillus* showed the potential to grow from low to high (20–2,000 mg L^−1^) levels of carbon, while some species of the genus *Exiguobacterium, Staphylococcus, Vibrio, Halomonas*, and *Pseudomonas* were unable to grow at a carbon concentration of 20 mg L^−1^. At 200 mg L^−1^ of carbon concentration, bacterial members *Pantoea, Alishewanella, Rheinheimera, Micrococcus*, and *Nitrincola* were unable to grow, indicating their true copiotrophic nature. Members of *Pantoea, Micrococcus, Nitrincola, Bacillus, Alishewanella*, and *Vibrio* showed the highest cell density (10^−8^ to 10^−10^), and it indicated that they are mostly dominating in Loner Lake sediment. Organisms in oligotrophic media showed low cell density compared to organisms of carbon-rich media either due to longer generation time or suppression of growth due to excessive carbon shock. It was observed that *Bacillus* and *Alishewanella* grew on both lower and higher carbon concentration, but there was a stark difference in their growth pattern; *Alishewanella* quickly attained exponential phase, but this phase was of shorter duration (~2.5 h) compared to *Bacillus*, which showed a long lag phase but also a prolonged exponential phase (6 h) when supplemented with low levels of C (Figure 2). In contrast, *Vibrio, Halomonas*, and *Exiguobacterium* were isolated only from the oligotrophic medium. All 49 isolates were able to grow at a 3% salt concentration. Except *for Bacillus marisflavi, Penibacillus hunanensis, Paenibacillus massiliensis, Lysinibacillus macrolides, Pseudomonas stutzeri, Pantoea septica, Alishewanella tabrizica*, and *Pseudomonas stutzeri*, all other isolates showed growth at 10% NaCl concentration and indicated that they are well-adapted for the saline condition of the lake. In addition, *Halomonas ventosae, Halomonas kenyensis, Paenibacillus massiliensis*, and *Staphylococcus gallinarum* grew at 20% NaCl concentration, although it is yet to be proven whether they are true halophiles or halotolerant. Evaluation of growth at different pH values indicated that all the strains showed growth between pH 4 and 10. While *Exiguobacterium, Nitrincola, Pseudomonas*, and some species of *Bacillus* did not show any growth at pH 7 but only at pH 10 and are likely to be true alkalifiles. Only a few strains showed growth between pH 4 and 10. Enzyme production was screened to relate the role of oligotrophic and copiotrophic bacteria in that particular ecological habitat (Lonar Lake). Enzyme-producing ability varied amongst the isolates, and protease production was more prevalent, followed by the urease. This observation corroborated with the previous observation that *Bacillus* was the dominating genus found in Lonar Lake sediment (Table 4) and has been widely reported for its ability to produce alkaline protease (Pathak and Deshmukh, 2012).

**Table 1:** Comparative study of bacterial load in oligotrophic and nutrient-rich mediums detected by MPN method and list of representative taxa cultivated from highest positive dilutions.

**Media**	**Highest positive dilution**	**Cell number** **(MPN count)**	**Isolates**
NB	10^−9^, 10^−10^, 10^−11^	2.15 × 10^10^	*Nitrincola*
LB	10^−7^, 10^−8^, 10^−9^	<24 × 10^8^	*Pantoea, Micrococcus, Alishewanella*
TSB	10^−9^, 10^−10^, 10^−11^	0.75 × 10^10^	*Bacillus*
R2A	10^−7^, 10^−8^, 10^−9^	4.62 × 10^8^	*Alishewanella*
RAVAN	10^−7^, 10^−8^, 10^−9^	1.5 × 10^8^	*Vibrio*
Synthetic	10^−6^, 10^−7^, 10^−8^	2.4 × 10^7^	*Halomonas*
NB-D	10^−3^, 10^−4^, 10^−5^	2.40 × 10^4^	*Dietzia, Exiguobacterium*
LB-D	10^−4^, 10^−5^, 10^−6^	0.93 × 10^5^	*Bacillus, Rheinheimera*
TSB-D	10^−4^, 10^−5^, 10^−6^	0.42 × 10^5^	*Staphylococcus, Alishewanella*
R2A-D	10^−3^, 10^−4^, 10^−5^	4.62 × 10^4^	*Vibrio*
RAVAN-D	10^−3^, 10^−4^, 10^−5^	2.4 × 10^4^	Not cultivated

NB-D, LB-D, and TSB-D = 1,000-fold diluted; R2A = 100-fold diluted; Ravan-D = 10-fold diluted.

**Table 2:** List of facultative oligotrophs and their physiological growth response on different levels of carbon, NaCl, and pH.

**Isolate No**.	**Similarity with closest relative (%)**	**Carbon (**≈**mg L**^**−1**^**)**			**% NaCl Tolerance**					**pH tolerance**		
		**20**	**200**	**2,000**	**3**	**5**	**10**	**15**	**20**	**4**	**7**	**10**
7	*Bacillus oceanisediminis* (99.8)	+	+	+	+	+	+	+	–	–	–	+
8	*Exiguobacterium aurantiacum* (100)	+	+	+	+	+	+	–	–	–	–	+
9	*Exiguobacterium himgiriensis* (95.8)	ND	ND	+	+	+	+	+	–	–	–	+
10	*Exiguobacterium aurantiacum* (100)	+	+	+	+	+	+	+	–	–	–	+
11	*Bacillus marisflavi* (100)	+	+	+	+	+	+	+	–	–	–	+
12	*Bacillus marisflavi* (99.4)	ND	+	+	+	–	–	–	–	–	+	+
22	*Exiguobacterium aurantiacum* (99.9)	+	+	+	+	+	+	–	–	–	–	+
23	*Bacillus clausii* (95.2)	+	+	+	+	+	+	–	–	–	+	+
24	*Dietzia cercidiphylli* (100)	+	+	+	+	+	+	–	–	–	+	+
25	*Staphyllococcus gallinarum* (100)	+	+	+	+	+	+	–	–	–	+	+
27	*S. gallinarum* (97.3)	ND	ND	+	+	+	+	+	+	+	–	+
30	*Bacillus altitudinis* (100)	+	+	+	+	+	+	–	–	–	+	+
32	*Enterobacter cancerogenus* (99.7)	+	+	+	+	+	–	–	–	+	+	+
33	*Vibrio metschnikovii* (98.2)	+	+	+	+	+	+	–	–	–	+	+
34	*Penibacillus hunanensis* (96.1)	+	+	+	+	+	–	–	–	–	+	+
35	*Paenibacillus massiliensis* (96.3)	ND	ND	+	+	+	–	+	+	–	+	+
36	*Paenibacillus massiliensis* (94.3)	ND	ND	+	+	+	–	–	–	–	+	+
37	*Bacillus cereus* (100)	+	+	+	+	+	+	+	–	–	+	+
38	*Bacillus safensis* (99.7)	+	+	+	+	+	+	+	–	–	+	+
39	*Bacillus thuringiensis* (96.6)	+	+	+	+	+	+	–	–	–	+	+
40	*Bacillus cereus* (100.0)	+	+	+	+	+	–	–	–	–	+	+
43	*Bacillus firmus* (99.4)	ND	ND	+	+	+	–	–	–	–	–	+
44	*Halomonas kenyensis* (98.8)	+	+	+	+	+	+	+	+	–	+	+
45	*Halomonas kenyensis* (98.8)	+	+	+	+	+	+	+	+	–	+	+
47A	*Lysinibacillus macrolides* (99.6)	+	+	+	+	+	–	–	–	–	+	+
47B	*Pseudomonas stutzeri* (99.6)	+	+	+	+	+	–	–	–	–	+	+

ND, no data.

**Table 3:** List of copiotrophic organism and their growth response on different levels of carbon, NaCl, and pH.

**Isolate No**.	**Similarity with closest relative (%)**	**Carbon (**≈**mg L**^**−1**^**)**			**% NaCl**					**pH**		
		**20**	**200**	**2,000**	**3**	**5**	**10**	**15**	**20**	**4**	**7**	**10**
1	*Pantoea septica* (99.78)	–	+	+	+	+	+	–	–	+	+	+
2	*Pantoea septica* (98.07)	–	+	+	+	+	–	–	–	–	+	+
3A	*Alishewanella tabrizica* (97.98)	–	+	+	+	+	–	–	–	–	+	+
3B	*Alishwanella tabrizica* (98.22)	–	+	+	+	+	–	–	–	–	+	+
4	*Rheinheimera longhuensis* (98.69)	–	+	+	+	+	+	–	–	+	+	+
6	*Micrococcus yunnanensis* (99.65)	–	+	+	+	+	+	–	–	–	+	+
13	*Rheinheimera longhuensis* (97.90*)*	–	+	+	+	+	+	–	–	–	+	+
14	*Micrococcus yunnanensis* (99.44)	–	+	+	+	+	+	+	–	–	+	+
17	*Nitrincola lacisaponensis* (99.71)	–	–	+	+	+	+	–	–	–	–	+
18	*Nitrincola lacisaponensis* (99.54)	–	–	+	+	+	+	–	–	–	–	+
19	*S. epidermidis* (100)	–	+	+	+	+	+	+	–	–	+	+
20	*S. epidermidis* (100)	–	+	+	+	+	+	+	–	+	+	+
21	*Exiguobacterium aurantiacum* (96.58)	–	–	+	+	+	+	+	–	–	–	+
28	*Alishewanella tabrizica* (98.18)	–	+	+	+	+	+	+	–	–	+	+
31	*Bacillus anthracis* (99.7)	ND	ND	+	+	+	+	–	–	+	+	+
41	*Vibrio metschnikovii* (98.64)	–	+	+	+	+	–	–	–	–	+	+
42	*Vibrio metschnikovii* (97.5)	–	+	+	+	+	–	–	–	–	+	+
46A	*Staphylococcus nepalensis* (98.57)	–	–	+	+	+	–	–	–	–	+	+
46B	*Pseudomonas stutzeri* (99.93)	–	–	+	+	–	–	–	–	–	–	+
46C	*Pseudomonas stutzeri* (99.93)	ND	ND	+	+	–	–	–	–	–	–	+
48	*Alishewanella solinquinati* (98.78)	–	–	+	+	+	–	–	–	–	+	+
49	*Halomonas ventosae* (97.79)	–	+	+	+	+	+	+	+	–	+	+
50	*Pantoea septica* (99.64)	–	+	+	+	+	+	–	–	–	+	+

**Table 4:** Doubling time (G) and growth rate (k) of selected facultative oligotrophs on different carbon levels.

**Strain ID**.	**Carbon** **(≈mg L^−1^)**	**Doubling Time G (h)**	**Growth Rate** **(k = 1/G** **(h^−1^)**
***Nitrincola*** strain NA10LO17	20	0.96	1.04
	200	2.00	0.50
	1,000	1.45	0.69
***Micrococcus*** strain LA9LO6	20	3.56	0.28
	200	1.33	0.75
	1,000	1.13	0.89
***Bacillus*** strain LAD3LO11	20	3.17	0.32
	200	1.38	0.73
	1,000	1.86	0.54
***Alishwanella*** strain NAD2LO24	20	15.08	0.07
	200	1.51	0.66
	1,000	2.67	0.37
***Vibirio*** strain R2A8LO28	20	7.67	0.13
	200	6.14	0.16
	1,000	4.02	0.25
***Bacillus*** strain TS9LO31	20	6.26	0.16
	200	1.78	0.56
	1,000	2.24	0.45
***Dietezia*** strain RAV5LO41	20	1.88	0.53
	200	1.49	0.67
	1,000	2.22	0.45

We also studied the effect of additional sources of nitrogen and phosphorous on the growth of facultative oligotrophs. In some cases, bacterial growth was enhanced after adding extra nitrogen, while excess phosphorous seemed to limit or deter a few isolates, but it did not affect the growth of other isolates. This is a known phenomenon that few organisms cease to grow in the presence of increased P- concentrations.

The screening of different sugars and glycerol as sources of carbon and energy showed that all the selected organisms grew satisfactorily on 50 mM dextrose. Out of seven, five showed almost similar ODs while the ODs of *Dietzia* and *Nitrincola* were higher than the other selected strains (Figure 1). Based on this observation, 50 mM dextrose was selected to study the growth kinetics. During our growth kinetics experiments, we were unable to detect any visible colonies on the agar plate supplemented with 20 mg L^−1^ carbon and could not determine their growth from the selected broth medium spectrophotometrically due to negligible optical density. The viability count was obtained using the CFU method on a high carbon medium to address this problem because all oligotrophs eventually adapt to higher carbon concentrations.

Furthermore, initially, we decided to take the CFU after every 2 h but did not get any colony due to immediate consumption of carbon from oligotrophic medium and entry of cells in death or viable but non-culturable (VBNC) stage. To address this, the sampling duration was curtailed to 30 min, which allowed determining the growth trend of organisms at low carbon concentration in an oligotrophic medium (Table 4). Our data also indicate that except *Nitrincola* the generation time of other strains shortened and the growth rate increased at higher carbon (200–1,000 mg L^−1^) (Figure 2), but it was almost similar at 200 and 1,000 mg L^−1^ carbon.

## Discussion

There is no commonly accepted definition for oligotrophic organisms specifying their exact carbon requirement (Poindexter, 1981; Schut et al., 1997; Hartke et al., 1998; Lee et al., 2021). According to Poindexter (1981), oligotrophs grow on a medium containing 1–15 mg of organic carbon L^−1^. Upon subsequent culturing, they become cultivable in nutrient-rich medium (Poindexter, 1981; Van Gemerden and Kuenen, 1984; Schut et al., 1997; Hartke et al., 1998; Gao and Wu, 2018). Fry (1990) defined oligotrophy in marine environments where the carbon requirement was <10 mg L^−1^. Hence, the carbon requirement of the oligotrophs may depend on the ecosystems, whether terrestrial or marine (Fry, 1990). In this study, the habitat, i.e., Lonar Lake, is a highly alkaline and saline soda lake. Because of its uniqueness, several studies related to geochemistry, cultivation, and characterization of microbial diversity from sediment and water samples of Lonar Lake have been carried out (Joshi et al., 2008; Antony et al., 2010, 2013, 2014; Surakasi et al., 2010; Paul et al., 2016; Bagade et al., 2020). However, there are no reports of oligotrophs/copiotrophic populations dominating the hypersaline and hyper-alkaline Lonar Lake ecosystem and their relation to geochemical features of the site, which may play cryptic roles in promoting them. In this study, we tried to elucidate whether k-strategist or r-strategist type of organisms dominate in hyper-alkaline and hypersaline habitats of Lonar Lake.

To quantify and differentiate the bacterial population growing at high and low levels of carbon in the sediment of Lonar Lake, MPN study was conducted. MPN study indicated that undiluted complex media like NB, LB, TSA, and R2A supported a maximum cell count of 10^−8^ to 10^−10^ per gram of wet weight of the sediment, while diluted complex media and other oligotrophic media used during this study supported only 10^−4^ to 10^−7^ cells per gram of wet weight. In addition, our result also suggests that the composition and concentration of media components play an essential role in selecting organisms from a habitat or while counting organisms using the MPN method. For instance, LB and NB components, though almost identical but owing to the double strength of LB *Pantoea, Micrococcus*, and *Alishewanella*, were favored while diluted NB promoted only *Nitrinicola*. Similar was the case with oligotrophic media like SGWM (promoted *Halomonas*), Ravan (promoted only *Vibrio*), and R2A (promoted *Alishewanella*) at higher dilution. Our observations indicated that though the genera *Nitrinicola* and *Bacillus* were dominant in the Lonar Lake they could not be manifested in TSB purely due to the media effect. Therefore, it may prove to be safer and wiser to include multiple media to get a fairer picture of cell count and dominance. Growth of certain organisms such as *Nitrinicola, Exiguobacterium, Staphylococcus, Pseudomonas*, and *Alishewanella* was deterred by carbon concentrations ≤ 200 mg L^−1^ and thus they may be considered true copiotrophs. The true oligotrophs that stopped growing ≥200 mg carbon L^−1^ were not found (Tables 2, 3). We also found that all the strains isolated using oligotrophic media could subsequently adapted to high carbon concentration and thus proved their ability to grow in complex carbon media. The lower cell count of these organisms in the MPN indicated their sustainability to high concentrations of carbon. However, due to the short generation time and faster growth rate of their competitor copiotrophs in the given milieu, these oligotrophs were kept at bay.

Based on our MPN results, we can predict that copiotrophy may dominate over oligotrophy in the Lonar Lake since the count of oligotrophic organisms (organisms in diluted medium) was lower than the number of copiotrophic (organisms growing in undiluted medium) organisms cultivated from the same sample. Physicochemical characterization of Lonar Lake in the past has revealed high Biological Oxygen Demand (BOD) and Chemical Oxygen Demand (COD) levels confirming high organic carbon facilitating eutrophic nutritional status. This is reflected in the MPN analyses favoring copiotrophs (Antony et al., 2010; Sharma et al., 2016).

The 20 isolates cultivated from oligotrophic media also show the ability to grow in the complex medium and can be classified as the second category of oligotrophs or facultative oligotrophs (Poindexter, 1981; Kuznetsov et al., 2003). Interestingly, we have reconfirmed earlier observations of Senechkin et al. (2010). These investigators deployed 200 different isolates, initially grown on oligotrophic media, to subsequently adapt and grow on highly nutritive media for 10 successive generations (accept two strains). Conversely, we also observed that six isolates obtained from undiluted rich media like MPN were unable to grow at carbon concentration lower than 200 mg L^−1^ and thus may prove to be true copiotrophs.

MPN data, along with cultivation, showed that *Bacillus* was predominant, followed by *Staphylococcus* and *Exiguobacterium*. As a matter of fact, the recovery of *Bacillus* from copiotrophic and oligotrophic media and its survivability in a wide spectrum of salinity and nutrients unequivocally establishes its ubiquity in various habitats (Lazar and Dumitru, 1973; Laiz et al., 2000, 2002a,b; Saiz-Jimenez and Laiz, 2000). A review of the past literature (Incerti et al., 1997; Joshi et al., 2008; Antony et al., 2010, 2013, 2014; Surakasi et al., 2010; Paul et al., 2016; Bagade et al., 2020) indicates that the closest relatives of our isolates such as *Nitrinicola, Alishwanella, Micrococcus, Halomonas*, and *Vibrio* were detected by culture-independent approach. This study demonstrated that they adapted to grow at higher alkalinity and salinity, and therefore they dominated in the Lonar Lake ecosystem.

Our results lend credence to the general notion that the cultivation of oligotrophs *sensu stricto* in low carbon regime is laborious and demands appropriate skills. Due to low cell biomass and optical density in the oligotrophic medium, it is difficult to visualize their growth with unaided eyes or spectrophotometrically, and they are left unattended by the usual isolation procedure. Furthermore, the data of culturing the strains on 20 mg L^−1^ carbon and the inability to form visible colonies after 2 h sampling indicated that despite the slow growth rate organisms utilized the available carbon in 2–3 generations and entered the death phase due to nutrient limitation, leading to the absence of visible colonies/detectable OD. Therefore, the strategy to supply low levels of nutrients continuously till visible OD was attained should be used for the successful cultivation of oligotrophs and also prevents contamination with copiotrophs, which only use high carbon-rich media. In addition, due to nutritional shock, true oligotrophs might not grow in a rich carbon medium when transferred directly from an oligotrophic environment.

Consequently, the pure oligotrophic strains could not be obtained as viable pure cultures. Therefore, it is recommended here that after successful cultivation or enrichment of cells in the liquid oligotrophic medium, they should be gradually exposed to high carbon concentrations (e.g., 50, 100, 200, 500, and 1,000 mg L^−1^) to obtain visible colonies on solid media plate and for their successful isolation and purification. The data on growth rate suggested that low as well as high C levels both limited the growth rate of the organisms, and we can obtain sufficient biomass without overloading the medium with extra carbon. These observations might pave the way for developing advanced protocols for the development of low-cost media and to add new link to the recent culturomics approaches. Media formulation similarities can be drawn with those from bioprocess engineering.

Life strategies and characteristic features of oligotrophic and copiotrophic organisms have been discussed in detail by Ho et al. (2017), which are very close to our findings that microbes cultivated using oligotrophic conditions get adapted to high nutrient conditions with gradual exposure to increasing concentrations of organic load in the medium (Ho et al., 2017). Using the computational model, Norris et al. (2021) demonstrated the occurrence of binding proteins is essential for oligotrophic life strategies (Newton and Shade, 2016; Norris et al., 2021). Weissman et al. (2021) estimated bacterial growth rates using genome sequences from cultured and uncultured representatives and concluded that oligotrophy and copiotrophy is a way of life strategies microorganisms adapt to sustain themselves in a particular habitat (Weissman et al., 2021). As rightly surmised, oligotrophic is not a taxonomic trait but a physiological adaptation and can be used as an attribute to capture the novel, not-yet-cultured slow-growing organisms (Bartelme et al., 2020). Oligotrophs can be a good source of industrial biomolecules, polysaccharides, and biofuels and can play a crucial role in waste management, but more in-depth studies are required to use them as a resource for these purposes (Porwal et al., 2008; Prakash et al., 2018; Lee et al., 2020).

## Conclusion

The majority of organisms in Lonar Lake sediment are essentially copiotrophs though facultative oligotrophs are also present. The dominance of copiotrophs indicates the eutrophic nature of the ecosystem. A distinct paucity of information appears to exist on the trophic organismic interactions from the peculiar soda lake and their overall contribution to the maintenance of the ecosystem health. Naturally, these aspects deserve deeper investigation. We showed that members of the same genus behave differently toward varied carbon regimes and may perform either as oligotrophic or copiotrophic suiting to their adaptation strategies of the habitat.

One can appreciate this new nutrition “economy classes” of oligotrophs that can become strong contenders to the traditional workhorses to address specific issues like low-cost production of industrial biomolecules and need extra attention for their cultivation and preservation. Our study opens new insights into the incipient contributions of “unculturable” or rarely culturable microbes in balancing the carbon and other nutrient cycles by seemingly easy crossovers from oligotrophy to copiotrophy in tune with the increasing carbon levels due to human interventions in the lake's ecosystem.

Most of the transcriptomics and metaproteomics functionality data hinge around rich media used for cultivation. This has left a yawning knowledge gap on the actual functionality of oligotrophs in the natural environs of soil and water. We also propose that the oligotrophs handled by us can be used as model systems for functionality studies in the natural oligotrophic conditions of soil and water on OMICS platforms.

## Data availability statement

The datasets presented in this study can be found in online repositories. The names of the repository/repositories and accession number(s) can be found at: NCBI - ON562524 - ON562538 and ON905511 - ON905529.

## Author contributions

Material preparation, data collection, and analysis were performed by YN, TG, AK, TP, and OP. The first draft of the manuscript was written by YN. Draft was edited by DhP, DeP, VR, and OP. Study conception and design was contributed by OP. All authors commented on previous versions of the manuscript and read and approved the final manuscript.

## Funding

This study was supported by the Grant (BT/Coord.II/01/03/2016) provided by the Department of Biotechnology (DBT) Govt. of India.

## Conflict of interest

The authors declare that the research was conducted in the absence of any commercial or financial relationships that could be construed as a potential conflict of interest.

## Publisher's note

All claims expressed in this article are solely those of the authors and do not necessarily represent those of their affiliated organizations, or those of the publisher, the editors and the reviewers. Any product that may be evaluated in this article, or claim that may be made by its manufacturer, is not guaranteed or endorsed by the publisher.
