# Supplementary material for: Oligotrophy vs. copiotrophy in an alkaline and saline habitat of Lonar Lake
Source: Front Microbiol. 2022 Aug 4;13:939984. doi: 10.3389/fmicb.2022.939984 (PMC9386271; doi:10.3389/fmicb.2022.939984)
Supplement: Supplementary file 1 [file Table_1.docx]

**Supplementary Table 1: Different enzyme production potentials of isolated strains**

| Isolate No. | Taxonomic identity | Enzyme Production | | | | | | |
| --- | --- | --- | --- | --- | --- | --- | --- | --- |
|  |  | 1 | 2 | 3 | 4 | 5 | 6 | 7 |
| 7 | *Bacillus oceanisediminis* | - | - | - | - | - | - | - |
| 8 | *Exiguobacterium aurantiacum* | - | + | - | - | - | - | - |
| 9 | *Exiguobacterium himgiriensis* | - | + | - | - | - | - | - |
| 10 | *Exiguobacterium aurantiacum* | - | + | - | - | - | - | - |
| 11 | *Bacillus marisflavi* | - | + | - | - | - | - | - |
| 12 | *Bacillus marisflavi* | - | + | - | - | - | - | - |
| 22 | *Exiguobacterium aurantiacum* | - | + | + | - | - | - | - |
| 23 | *Bacillus clausii* | - | + | - | - | - | - | - |
| 24 | *Dietzia cercidiphylli* | - | - | - | - | - | - | - |
| 25 | *Staphyllococcus gallinarum* | - | - | - | - | - | - | - |
| 27 | *S. gallinarum* | - | + | - | - | - | - | - |
| 30 | *Bacillus altitudinis* | + | + | - | - | - | + | - |
| 32 | *Enterobacter cancerogenus* | - | - | - | - | - | - | - |
| 33 | *Vibrio metschnikovii* | - | + | - | - | - | + | - |
| 34 | *Penibacillus hunanensis* | - | + | - | - | - | + | - |
| 35 | *Paenibacillus massiliensis* | - | + | - | - | - | + | - |
| 36 | *Paenibacillus massiliensis* | + | - | - | - | - | + | - |
| 37 | *Bacillus cereus* | - | + | - | - | + | - | - |
| 38 | *Bacillus safensis* | - | + | - | - | - | - | - |
| 39 | *Bacillus thuringiensis* | - | + | - | - | + | - | - |
| 40 | *Bacillus cereus* | - | + | - | - | - | - | - |
| 43 | *Bacillus firmus* | - | - | - | - | - | - | - |
| 44 | *Halomonas kenyensis* | - | - | - | - | - | - | - |
| 45 | *Halomonas kenyensis* | + | - | - | - | - | - | - |
| 47A | *Lysinibacillus macroides* | + | - | + | - | - | - | - |
| 47B | *Pseudomonas stutzeri* | + | - | + | - | - | - | - |
| 1 | *Pantoea septica* | - | - | - | + | - | - | - |
| 2 | *Pantoea septica* | - | + | - | - | - | - | - |
| 3A | *Alishewanella tabrizica* | - | + | - | - | - | - | - |
| 3B | *Alishwanella tabrizica* | - | + | - | - | - | - | - |
| 4 | *Rheinheimera longhuensis* | - | - | - | - | + | - | - |
| 6 | *Micrococcus yunnanensis* | - | - | + | - | - | - | - |
| 13 | *Rheinheimera longhuensis* | - | + | - | - | - | - | - |
| 14 | *Micrococcus yunnanensis* | - | - | + | - | - | - | - |
| 17 | *Nitrincola lacisaponensis* | - | - | + | - | - | - | - |
| 18 | *Nitrincola lacisaponensis* | - | - | - | - | - | - | - |
| 19 | *S..epidermidis* | - | + | + | - | - | - | - |
| 20 | *S. epidermidis* | - | + | + | - | + | - | - |
| 21 | *Exiguobacterium aurantiacum* | - | + | - | - | - | - | - |
| 28 | *Alishewanella tabrizica* | + | + | - | - | - | - | - |
| 31 | *Bacillus anthracis* | - | + | - | - | - | - | - |
| 41 | *Vibrio metschnikovii* | - | + | - | - | - | - | - |
| 42 | *Vibrio metschnikovii* | - | + | - | - | - | - | + |
| 46A | *Staphylococcus nepalensis* | - | + | + | - | - | - | - |
| 46B | *Pseudomonas stutzeri* | - | - | - | - | - | - | - |
| 46C | *Pseudomonas stutzeri* | - | - | - | - | - | - | - |
| 48 | *Alishewanella solinquinati* | - | - | + | - | - | - | - |
| 49 | *Halomonas ventosae* | - | - | + | - | - | - | - |
| 50 | *Pantoea septica* | - | - | + | + | - | - | - |

+, Positive test; -, Negative test

1, DNase; 2, Protease; 3, Urease; 4, Phoshatase; 5, Cellulase; 6, Amylase; 7, Gelatinase.
